# Ruthenium-loaded mesoporous silica as tumor microenvironment-response nano-fenton reactors for precise cancer therapy

**DOI:** 10.1186/s12951-021-00848-x

**Published:** 2021-04-07

**Authors:** Dongdong Sun, Zekun Wang, Pu Zhang, Chenyang Yin, Jingyuan Wang, Yu Sun, Ying Chen, Weiyun Wang, Baoliang Sun, Cundong Fan

**Affiliations:** 1 School of Life Sciences, Anhui Agricultural University, Hefei, 230036 China; 2 Department of Cardiovascular Medicine, Taian City Central Hospital, Taian, 271000 Shandong China; 3 Department of Neurology, Second Affiliated Hospital; Key Lab of Cerebral Microcirculation in Universities of Shandong, Shandong First Medical University & Shandong Academy of Medical Sciences, Taian, 271000 Shandong China

**Keywords:** Tumor microenvironment, Mesoporous silica, Nano-Fenton reactors, Cancer precise therapy

## Abstract

**Background:**

Nano-Fenton reactors as novel strategy to selectively convert hydrogen peroxide (H_2_O_2_) into active hydroxyl radicals in tumor microenvironment for cancer therapy had attracted much attention. However, side effects and low efficiency remain the main drawbacks for cancer precise therapy.

**Results:**

Here, ruthenium-loaded palmitoyl ascorbate (PA)-modified mesoporous silica (Ru@SiO_2_-PA) was successfully fabricated and characterized. The results indicated that Ru@SiO_2_-PA under pH6.0 environment displayed enhanced growth inhibition against human cancer cells than that of pH7.4, which indicated the super selectivity between cancer cells and normal cells. Ru@SiO_2_-PA also induced enhanced cancer cells apoptosis, followed by caspase-3 activation and cytochrome-c release. Mechanism investigation revealed that Ru@SiO_2_-PA caused enhanced generation of superoxide anion, which subsequently triggered DNA damage and dysfunction of MAPKs and PI3K/AKT pathways. Moreover, Ru@SiO_2_-PA effectively inhibited tumor spheroids and tumor xenografts growth in vivo by induction of apoptosis. The real-time imaging by monitoring Ru fluorescence in vitro and in vivo revealed that Ru@SiO_2_-PA mainly accumulated in cell nucleus and tumor xenografts. Importantly, Ru@SiO_2_-PA showed no side effects in vivo, predicting the safety and potential application in clinic.

**Conclusions:**

Our findings validated the rational design that Ru@SiO_2_-PA can act as novel tumor microenvironment-response nano-Fenton reactors for cancer precise therapy.

**Graphic Abstract:**

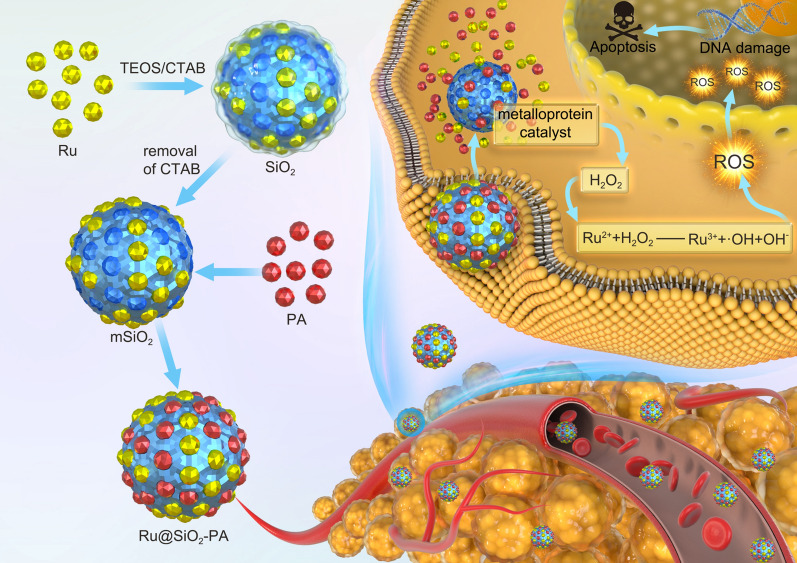

## Introduction

Normal cells usually maintain a redox balance between the generation of reactive oxygen species (ROS) and ROS elimination [1]. Disorder of redox homeostasis may result in damage to biological molecules, leading to inflammation and even tumorigenesis [2, 3]. However, cancer cells usually show high level oxidative stress compared with that of normal tissues, which plays key role in driving tumor proliferation and tumor development [4, 5]. Ironically, cancer cells are fragile to overproduced ROS because of the deficiency of ROS-depleting enzyme systems in tumor regions [6–8]. Overproduction of ROS can damage kinds of biological molecules and induce oxidative damage, which is accepted as an effective way for cancer therapy [2, 3]. Accumulated studies confirmed that ROS-mediated oxidative damage and apoptotic cell death of cancer cells contribute to chemotherapy, radiotherapy, and photodynamic of tumors [9–11]. Therefore, design of anticancer drugs with amplified oxidative stress in tumor regions to preferentially kill cancer cells has been accepted as novel strategy for precise cancer therapy, named as oxidation therapy.

Oxidation therapy refers to delivery of exogenous ROS or ROS-generating agents to tumor regions, or inhibition of ROS-depleting enzymes [7, 12], and now a large number of ROS-generating molecular have been found for nano-Fenton reactors to increase the ROS level in tumor regions [13–15]. Nano-Fenton reactor carrying H_2_O_2_-generating agent and catalyst can convert H_2_O_2_ into high cytotoxic hydroxyl radicals in tumor acidic microenvironment to selectively kill cancer cells and has been well studies [16–19]. High metabolic level of tumor tissue mainly depends on anaerobic glycolysis to provide energy, which produces a large amount of lactobionic acid [20]. In order to maintain a neutral cytoplasmic environment and avoid acidosis, tumor cells can produce a large amount of H^+^ and pump them out of the cell by ion exchanger, which results in the acidic extracellular tumor microenvironment [21]. Accumulated studies have confirmed that vitamin C or its derivate, such as palmitoyl ascorbate (PA), usually displays antioxidant activity at physiological concentration. However, vitamin C and its derivate usually act as a prooxidant therapeutic agent at pharmacologic concentration in therapy of cancer and infections [22]. Vitamin C (VC) and its derivates can be oxidized by a metalloprotein in tumor acidic microenvironment and yield tumoricidal H_2_O_2_, which is often employed as H_2_O_2_-generating agent in Nano-Fenton reactors [23–25]. Ferrocene as the catalyst of Nano-Fenton reactors undergoes oxidation to produce Fe^2+^, which can convert H_2_O_2_ into hydroxyl radicals [10, 26, 27]. So far, kinds of nano-Fenton reactors have been designed and achieved promising outcomes in combating human cancer. For instance, Ma et al.explored an enhanced cisplatin chemotherapy by iron oxide nanocarrier-mediated generation of highly toxic ROS in tumor regions through Fenton reaction [28]. Yin et al. reported that PEG-modified PA-integrated hybrid micelles showed amplified tumor oxidative stress for cancer oxidation therapy [29]. Kwon et al. constructed H_2_O_2_-generating benzoyloxycinnamaldehyde (BCA) and ferrocene catalyst, and the result indicated that PolyCAFe micelles can act as a new nano-fenton reactors with amplified oxidative stress in tumor regions for cancer therapy [18]. However, side effects and low efficiency remain the drawbacks in limiting its development. In the present study, ruthenium-loaded palmitoyl ascorbate (PA)-modified mesoporous silica (Ru@SiO_2_-PA) as a novel nano-Fenton reactor was successfully fabricated and characterized. H_2_O_2_ was generated in the tumor acidic microenvironment in the presence of PA, and Ru^2+^ as the catalyst can catalyze H_2_O_2_ into high active hydroxyl radicals [23]. The results revealed that Ru@SiO_2_-PA showed selectively growth inhibition against cancer cells in vitro and in vivo by triggering ROS-mediated oxidative stress and regulating multiple signal pathways. Meanwhile, Ru@SiO_2_-PA displayed less side effects and good biocompatibility in vitro and in vivo. Our findings validated the rational design that ruthenium-loaded PA-modified mesoporous silica (Ru@SiO_2_-PA) can act as novel tumor microenvironment-response nano-Fenton reactors for cancer precise therapy.

## Experimental section

### Chemicals

Tris (2,2-Bipyridyl) Ruthenium (II) Chloride Hexahydrate, Ethyl orthosilicate (TEOS), cetyltrimethylammonium chloride (CTAC), aminopropyltriethylaminosilane (APTES), ammonium nitrate and L-ascorbyl palmitate (PA) were all purchased from Aladdin (Shnaghai, China). Hiazolyl blue tetrazolium bromide (MTT), Dihydroethidium (DHE) probes, glutathione (GSH), and other agents were all purchased from Sigma-Aldrich. BCA kit, Giemsa staining and Annexin V was purchased from Beyotime (Beijing, China). Dulbecco's modified eagle medium (DMEM), fetal bovine serum (FBS) and penicillin–streptomycin was purchased from Invitrogen. All antibodies and inhibitors used in this study were bought from Cell Signaling Technology (CST, Beverly, MA, USA). All solvents were obtained with high performance liquid chromatography (HPLC) grade. Ultrapure MilliQ water (18.25 MW) was used in all experiments.

### ***Synthesis of Ru@SiO***_***2***_***-PA***

Briefly, 10 mg of ruthenium complex was dissolved in a mixed solution of 50 ml of deionized water and 60 ml of absolute ethanol. Under constant stirring, 3.5 ml of ammonia water and 0.12 g of CTAC were added to the reaction system. After stirring for 5 min, 200 μl APTES was added, and then 1 ml of TEOS (0.25 ml/min) was added dropwise. After stirring for 6 h, obtain the precipitate by centrifugation (10,000 rpm, 10 min), and washed three times with absolute ethanol and pure water, respectively. The pellet was washed three times with ethanol and water and then dispersed in a solution of NH_4_NO_3_ (50 ml, 5 mg/ml) in ethanol, and the mixture was stirred under reflux at 60 °C for 5 h to remove the CTAC template by an ion exchange method. The product was obtained by centrifugation (10,000 rpm, 10 min), washed three times with methanol and pure water, and then dried in a vacuum environment at 40 °C for 24 h to obtain Ru@SiO_2_ shell-core nanospheres.

### *Loading and *in vitro* release of PA*

Ru@SiO_2_ dry powder (5 mg) was added to 10 ml PA ethanol solution (2 mg/ml), and was stirred for 24 h at room temperature. The excess PA was removed by centrifugation (10,000 rpm, 10 min), and washed three times with anhydrous ethanol, and dried under vacuum to obtain Ru@SiO_2_-PA nanospheres. All supernatants were collected and appropriately diluted, and the absorbance was measured at 246 nm by a UV–visible spectrophotometer to calculate the amount of PA payload in Ru@SiO_2_ nanospheres. Drug loading of the Ru@SiO_2_-PA = (weight of PA loaded into Ru@SiO_2_) / (total weight of Ru@SiO_2_-PA). The in vitro release of PA from Ru@mSiO_2_ was investigated using the previously reported dialysis method. In short, 1 ml of Ru@SiO_2_-PA (1 mg/ml) was added to a dialysis bag (molecular cutoff of 10 KD) and placed in 19 ml of PBS containing 0.1% Tween 80 (pH = 7.4/6.0), collect and replace the release medium every 2 h, and quantify the PA in the medium by an ultraviolet spectrophotometer. In addition, PA was dissolved in 0.1% Tween 80 in PBS (pH = 7.4), and the release of PA solution was assessed based to the above method to determine the effect of the dialysis bags on the diffusion of chlorogenic acid molecules. All experiments were repeated at least 3 times.

### ***Characterization of Ru@SiO***_***2***_***-PA***

The shape and surface characteristics of Ru@SiO_2_-PA nanospheres were analyzed by transmission electron microscope (TEM; HT7700; Hitachi, Japan) and scanning electron microscope (SEM; S-4800; Hitachi, Japan). Energy dispersive X-ray spectrometer (EDS, Oxford, X-Max N 150) is used for elemental analysis. The dynamic light scattering (DLS) and zeta potentials measurements were used for characterization of NPs optical properties and sizes on a Zeta-PALS (Brookhaven) instrument. X-ray photoelectron spectroscopy (XPS) was used to study the elemental composition and valence of Ru@mSiO_2_-PA. X-ray diffraction (XRD; XRD-6100; Shimadzu, Japan) is used to study the crystal structure of nanospheres. The UV–Vis spectrum (Scinco Co., Korea) and Fourier transform infrared spectrum (FTIR; Nicoletteis50; Thermo Fisher Science, USA) were used to determine the synthesis of nanospheres.

H_2_O_2_ production of PA in Dulbecco’s modified Eagle’s medium (DMEM) with 10% FBS was measured using a dissolved oxygen meter in the presence of catalase. Briefly, PA at final concentrations of 3, 6, 12 or 24 μg/ml was added to DMEM containing 10% FBS and incubated at room temperature. At predetermined time intervals, 10 ml aliquots of the culture medium were removed, then 200 μl of 1000 units of catalase solution were added, and an O_2_ sensor was used to evaluate the pro-oxidation of PA.

^.^OH radicals were detected using disodium terephthalate as a capture agent. Briefly, 200 μl of disodium terephthalate solution (50 mM) and 200 μl of PBS, PA (6 μg/ml), Fe_3_O_4_@mSiO_2_ (14 μg/ml) or Fe_3_O_4_@mSiO_2_-PA (20 μg/ml) were added to1.6 ml of DMEM containing 10% FBS. The solution was subjected to fluorescence tracing using a fluorescence spectrometer (*Ex* = 310 nm, *Em* = 425 nm).

### Cell culture and cell viability

HepG2 human hepatocarcinoma cells, MCF-7 human breast carcinoma cells, and SGC-7901 human gastric carcinoma cells were obtained from ATCC company and cultured with DMEM medium supplemented with 10% fetal bovine serum and penicillin (100 units/ml), and streptomycin (50 units/ml) at 37 ℃ under 5% CO_2_ atmosphere. HUVECs human umbilical vein endothelial cells were cultured with endothelial culture medium and were employed as the normal cells. Medium was adjusted into pH6.0 by HCl to stimulate the acidic tumor microenvironment. The normal medium (pH7.4) was set as the neutral microenvironment. Cells (10^4^ cells/well) were seeded in 96-well plate and cultured under pH6.0 and pH7.4 medium for 24 h. Cells were treated with 2.5–20 μg/ml Ru@SiO_2_ or Ru@SiO_2_-PA for 72 h. Cells viability was detected by MTT assay. All experiments were repeated at least 3 times. Cells viability was expressed as percentage of control (as 100%).

### Detection of cells apoptosis

SGC-7901 cells seeded in 2-cm culture plate were exposed to 2.5–20 μg/ml Ru@SiO_2_ or Ru@SiO_2_-PA for 12 h. Cells after treatment were incubated with annexin V probe for 15 min in darkness. Then cells were washed with PBS, and the early cells apoptosis was imaged by a fluorescent microscope (magnification, 200×).

### Measurement of caspase-3 activity

SGC-7901 cells seeded in 9-cm culture plate were exposed to 2.5–20 μg/ml Ru@SiO_2_ or Ru@SiO_2_-PA for 72 h. Cells after treatment were washed, collected by centrifugation, and the total protein was extracted and quantified by BCA kit. 100 μg/well of protein was added into 96-well plate and incubated with specific Ac-DEVD-AMC substrate for 40 min in darkness. The caspase-3 activity was detected by fluorescent microreader with the 380 excitation and 440 nm emission.

### Western blotting

SGC-7901 cells seeded in 9-cm culture plate were exposed to 2.5–20 μg/ml Ru@SiO_2_ or Ru@SiO_2_-PA for 72 h. Cells after treatment were washed, collected by centrifugation, and the total protein was extracted and quantified by BCA kit. 40 μg/lane protein was loaded and separated by electrophoresis. Then, protein was transferred onto a nitrocellulose membrane, and blocked by 5% non-fat milk in TBS buffer for 1 h. Then, the membranes were washed and incubated with primary antibodies overnight at 4℃. Membranes subsequently were washed and incubated specific second antibodies for 2 h at room temperature. Then, the membranes were washed and the target proteins were detected on by chemiluminescence reagent under a Bio-Rad image system.

### Inhibitory effects on tumor spheroids

Ru@SiO_2_-PA-induced anticancer efficiency was also evaluated in 3-dimensional (3D) multicellular tumor spheroids as described previously [18]. Briefly, SGC-7901 cells (6 × 10^5^ cells/well) were seeded in ultra-low attachment 6-well plate and cultured for 2 days. Then, tumor spheroids were treated with 2.5–20 μg/ml Ru@SiO_2_ or Ru@SiO_2_-PA for 72 h. After incubation, the tumor spheroids were observed by a fluorescent microscope (magnification, 100 ×). The quantitative analysis of tumor spheroids volume was conducted to evaluate Ru@SiO_2_-PA-induced anticancer efficiency against SGC-7901 cells. All experiments were repeated at least 3 times.

### Examination of oxidative stress

The status of oxidative stress in SGC-7901 cells was monitored by measuring the accumulation of superoxide anion with DHE probe. Briefly, SGC-7901 cells seeded in 2-cm plate were pre-loaded with 10 μM DHE probe for 15 min in darkness, and cells were washed and exposed to 10 μg/ml Ru@SiO_2_ or Ru@SiO_2_-PA for 10–120 min. Then, the real-time generation of superoxide anion (red fluorescence) was imaged under a fluorescent microscope (magnification, 100 ×). The real-time generation of superoxide anion was also examined in tumor spheroids. Briefly, SGC-7901 cells (6 × 10^5^ cells/well) were seeded in ultra-low attachment 6-well plate and cultured for 5 days. Then, SGC-7901 tumor spheroids were pre-incubated with DHE probe and washed, and treated 20 μg/ml Ru@SiO_2_ or Ru@SiO_2_-PA for 10–120 min. The real-time generation of superoxide anion was imaged under a fluorescent microscope (magnification, 100 ×).

### Anticancer activity in vivo

Ru@SiO_2_-PA-induced anticancer activity in vivo was examined in nude mice bearing SGC-7901 tumor xenografts. Briefly, SGC-7901 cells (10^7^ cells) were subcutaneously injected into the nude mice. After 7-days tumor growth, mice were given 2.5 and 5 mg/kg Ru@SiO_2_-PA by tail intravenous injection every other day for 21 days. The real-time tumors volume change was monitored. After treatment, nude mice bearing SGC-7901 tumor xenografts were imaged, and the final tumors were collected and imaged. The tumors weight were measured and quantified. Part of tumor tissue was used for western blot analysis.

### Biodistribution in vitro and in vivo

Biodistribution Ru@SiO_2_-PA was firstly examined in vitro. Briefly, SGC-7901 cells seeded in 2-cm plate were treated with 2.5 μg/ml Ru@SiO_2_-PA for 3–12 h. The real-time biodistribution of Ru@SiO_2_-PA in vitro were imaged by measure the Ru fluorescence (magnification, 200 ×). The in vivo biodistribution of Ru@SiO_2_-PA was monitored by real-time imaging in nude mice. Briefly, Ru@SiO_2_-PA (10 mg/kg) was given by tail vein injection, and real-time imaging of Ru fluorescence at 1, 2 and 3 h was detected by in vivo fluorescence imaging system (Caliper Perkin Elmer).

### Analysis of pathology and hematology

Safety of Ru@SiO_2_-PA in vivo was evaluated in nude mice by analysis of pathology and hematology. Briefly, Nude mice after treatment were all given euthanasia, and main organs (heart, liver, spleen, lung, kidney and brain) were collected, cut into 4 μM slices and stained by H&E for histopathological examination under a microscope (magnification, 100×). Moreover, blood from nude mice or nude mice bearing SGC-7901 tumor xenografts was collected, and blood glucose (GLU), cholesterol (CHOL), serum creatinine (CREA), albumen (ALB), globulin (GLB) and lactate dehydrogenase (LDH) were all detected by ELISA methods to evaluate the health of kidney, liver and heart. All experiments were repeated at least 3 times. Health nude mice without tumor xenografts were used as the positive control group. Nude mice with tumor xenografts were used as the blank.

### Statistical analysis

All experiments were done at least for three times. Data were expressed as mean ± SD. Statistical analysis was assayed by SPSS 13.0 (SPSS, Inc.). Statistical significance was analyzed by one-way ANOVA followed by a Dunnett’s or Tukey’s post-hoc test. *P < 0.05 vs. blank, **P < 0.01 vs. blank. Bars with different letters indicate the Statistical significance at P < 0.05 level.

## Results and discussion

### Synthesis and characterization of Ru@SiO_2_-PA

**Fig. 1:**
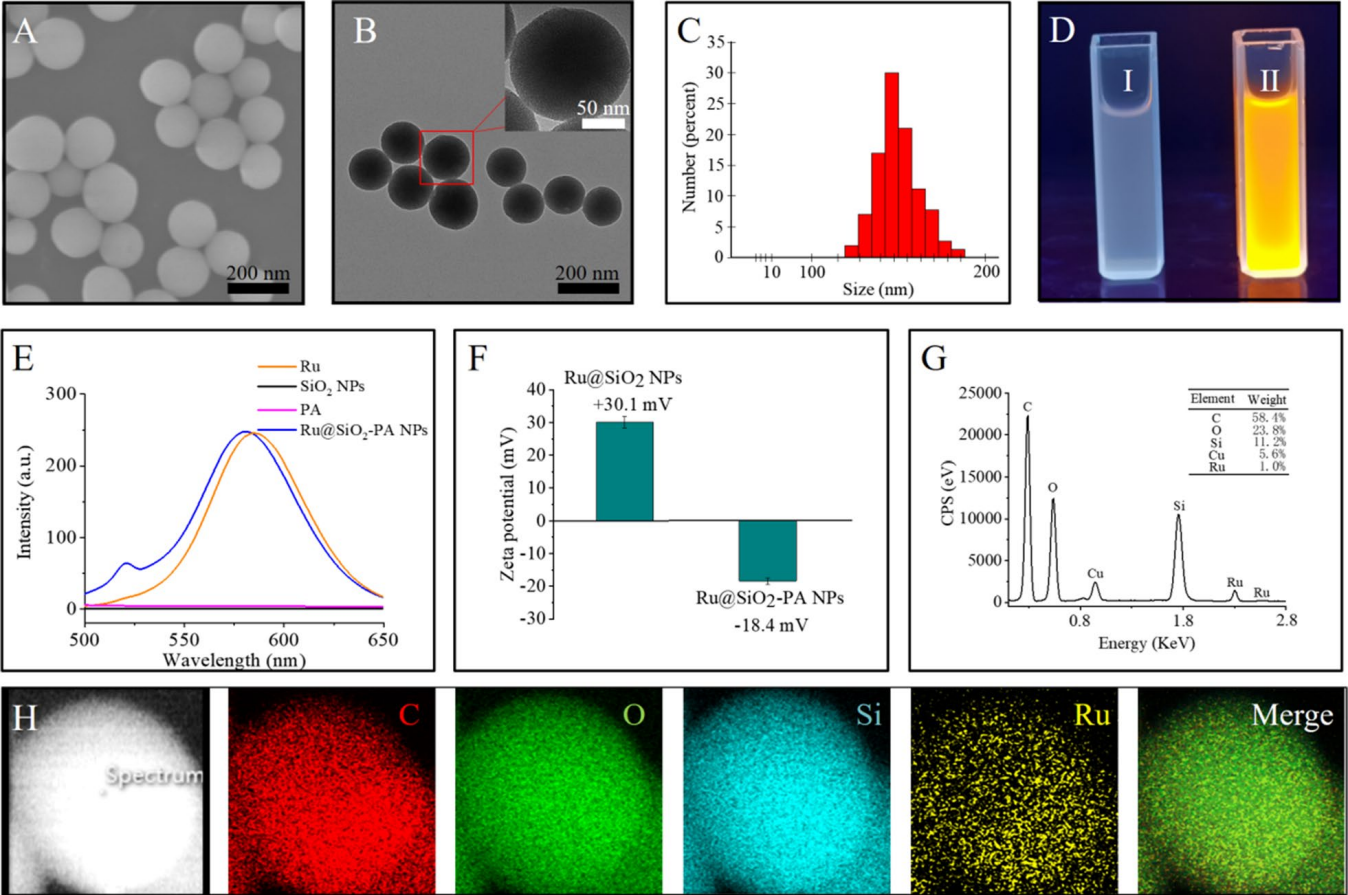
Synthesis and morphology of Ru@SiO_2_-PA. **a** SEM images and TEM images **b** of Ru@SiO_2_-PA. **c** Hydrodynamic diameters and distributions of Ru@mSiO_2_-PA. **d** Photographs of 1 mg/ml SiO_2_-PA (I) and Ru@SiO_2_-PA (II) under ultraviolet light. **e** Fluorescence emission spectra of PA, SiO_2_, Ru and Ru@SiO_2_-PA. **f** Variation of zeta-potentials of Ru@SiO_2_ and Ru@SiO_2_-PA during the coating process. **g** EDS spectrum of Ru@SiO_2_-PA. **h** Element mapping of Ru@SiO_2_-PA

SEM and TEM image showed that relatively homogeneous nanostructures had been successfully prepared (Fig. 1a, b). As shown in Fig. 1c, Ru@SiO_2_-PA has a uniform size, and the average diameter of the nanospheres was 140 ± 15 nm. As shown in Fig. 1d, the synthesized Ru@SiO_2_-PA could be uniformly dispersed in water and exhibited fluorescent properties under ultraviolet light, while PA and SiO_2_-PA did not exhibit any fluorescent properties (Fig. 1e). The zeta potential of the Ru@SiO_2_ dissolved in PBS was about + 30.1 mV. Those of the Ru@SiO_2_ coated with PA turned to be -18.4 mV (Fig. 1f). The elemental mapping images confirm that C, O, Si and Ru elements were distributed in the Ru@SiO_2_-PA homogeneously (Fig. 1g, h).

**Fig. 2:**
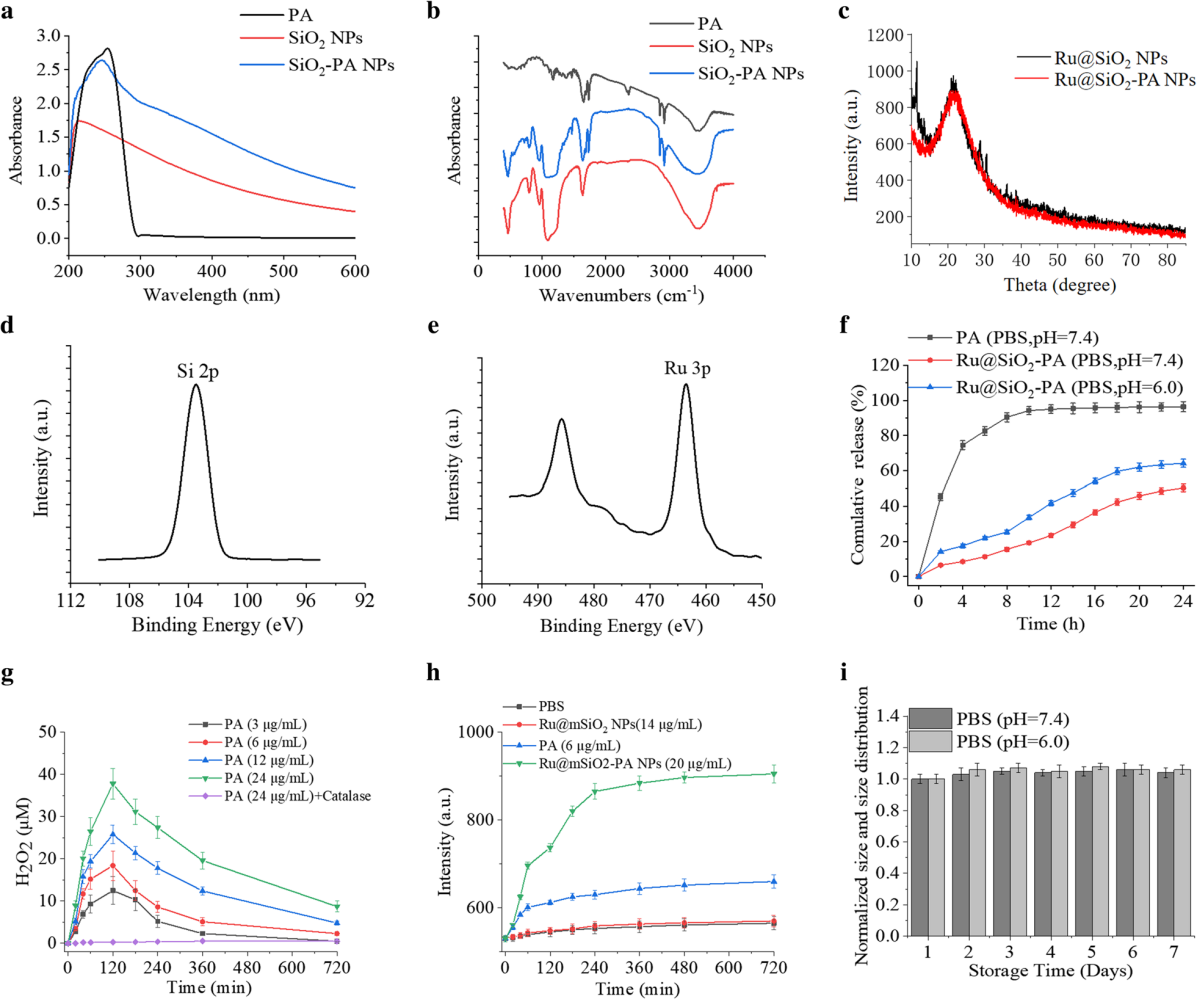
Characterization of Ru@SiO_2_-PA. **a** UV–vis absorption spectra of PA, SiO_2_ and SiO_2_-PA. **b** FTIR spectrum of PA, **c** XRD spectrum of Ru@SiO_2_-PA. SiO_2_ and SiO_2_-PA. Si^2P^ **d** and Ru^3P^ **e** XPS spectrum of Ru@SiO_2_-PA. **f** Relative PA release of Ru@SiO_2_-PA at pH = 7.4/6. **g** Cumulative H_2_O_2_ production in DMEM medium (10% serum) in the presence of PA. **h** Fluorescence intensity change of 2-hydroxyterephthalic acid as a function of time after incubation with PA (6 μg/ml), Fe_3_O_4_@mSiO_2_ (14 μg/ml), or Fe_3_O_4_@mSiO_2_-PA (20 μg/ml) in 10% serum-containing medium. (I) The relative size change of Ru@SiO_2_-PA at different storage time (1–7 days)

To confirm the formation of SiO_2_-PA, UV–vis Spectra assays were performed. Figure 2a shows PA has characteristic absorption peaks at 246 nm. Similar results were observed for SiO_2_-PA. The FTIR patterns of SiO_2_ show typical Si–O-Si tensile vibration peaks at 1070 cm^−1^ and 960 cm^−1^. PA showed stretching vibration peaks of -CH_2_ at 2851 cm^−1^ and 2917 cm^−1^, similarly appeared in SiO_2_-PA (Fig. 2b). The crystal structure of Ru@SiO_2_ and Ru@SiO_2_-PA was examined by XRD pattern (Fig. 2c). The two show a high degree of similarity in the spectrum, and there are obvious dispersion peaks at 20°—25° of 2θ, which was the standard characteristic peak of the typical amorphous structure SiO_2_. These results indicate that Ru@SiO_2_-PA was successfully synthesized. XPS analysis was used to study the surface composition and chemical bond state, which can provide information on the interaction between SiO_2_ and Ru. Figure 2d shows the Si2p spectrum, the peak at 102.6 eV was consistent with the previously reported binding energy of silica. Figure 2e shows the Ru3p spectrum, the two peaks of Ru3p_1/3_ and Ru3p_2/3_ are located at 484.5 eV and 462.4 eV, respectively. According to the standard curve of PA, the drug loading of Ru@SiO_2_-PA is calculated to be 30.7%. As shown in Fig. 2f, the cumulative release amount of the PA at pH = 7.4 was quite small within 24 h. However, due to the increased solubility of PA under acidic conditions, the release rate of the drug becomes faster at pH = 6.0, and the cumulative release of the drug was as high as 60.8%. This pH-dependent release profile was more beneficial for tumor therapy. Due to the oxidation reaction between PA and certain metal ion-containing proteins, H_2_O_2_ is effectively produced in a PA concentration-dependent manner. At the test concentration, the concentration of H_2_O_2_ reached its peak in about 120 min (Fig. 2g). The increased concentration provides the necessary H_2_O_2_ for the Fenton reaction to produce highly active •OH radicals. In addition, we also evaluated the production of OH radicals in vitro. As shown in Fig. 2h, the fluorescence intensity of only Ru@SiO_2_ and PBS remained almost constant. In sharp contrast, the fluorescence intensity of Ru@SiO_2_-PA increases rapidly at the equivalent concentration. In conclusion, the results confirmed that PA can produce H_2_O_2_ in serum-containing DMEM medium, and catalyze the production of a large number of •OH free radicals in the presence of Ru@SiO_2_, which can effectively destroy cancer cells. The storage stability of Ru@SiO_2_-PA suspended in aqueous and PBS solutions are illustrated in Fig. 2i. The size of the nanomaterials did not change significantly within 7 days, which suggests that the Ru@SiO_2_-PA are stable enough for further investigation.

### Ru@SiO_2_-PA selectively inhibits cancer cells growth

**Fig. 3:**
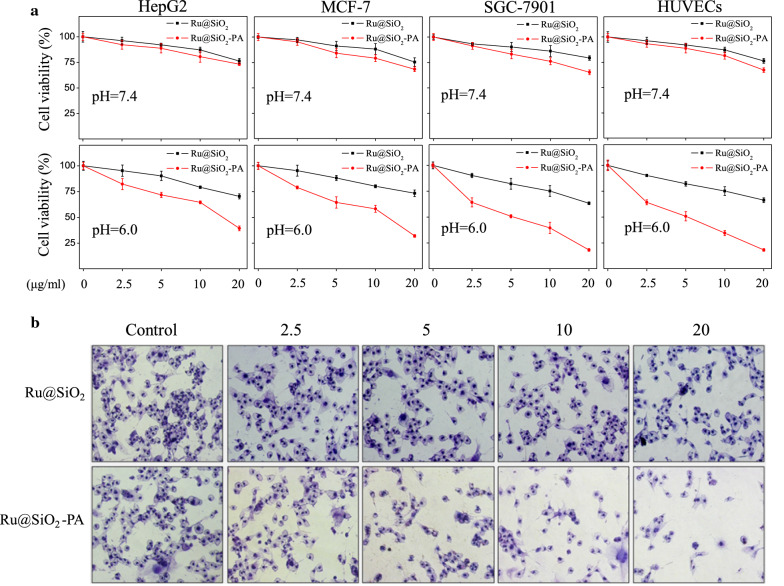
Ru@SiO_2_-PA selectively inhibited cancer cells growth. **a** Ru@SiO_2_-PA induced pH-response cells killing between cancer cells and normal cells. Cancer cells (MCF-7, HepG2 and SGC-7901) and normal cells (HUVECs) seeded in 96-well plate were cultured with pH 6.0 and pH 7.4 complete medium, and exposed to 2.5, 5, 10 and 20 μg/ml Ru@SiO_2_ or Ru@SiO_2_-PA for 72 h. Cells viability was detected by MTT assay. **b** Phase contrast of cells morphology. SGC-7901 cells after treatment were stained by Giemsa solution, and cells morphology was observed by a phase microscope (magnification, ×200)

Increased evidences have confirmed that nano-Fenton reactors showed novel anticancer potential in tumor acidic microenvironment [16–18]. Herein, Ru@SiO_2_-PA-induced tumor microenvironment-response anticancer activity was firstly examined in different pH environment. As shown in Fig. 3a, Ru@SiO_2_-PA under pH 6.0 environment significantly inhibited cancer cells growth of HepG2, MCF-7 and SGC-7901 with a dose-dependent manner. Ru@SiO_2_-PA under pH 7.4 environment only slightly inhibited cancer cells growth of HepG2, MCF-7 and SGC-7901. For instance, Ru@SiO_2_-PA (5, 10 and 20 μg/ml) under pH 7.4 environment only inhibited SGC-7901 cells viability from 100% (control) to 80.1%, 75% and 68.2%, respectively. However, Ru@SiO_2_-PA (5, 10 and 20 μg/ml) under pH 6.0 environment dose-dependently inhibited SGC-7901 cells viability from 100% (control) to 51.2%, 37.2% and 17.5%, respectively. Ru@SiO_2_ under pH 7.4 and pH 6.0 environment both caused slight cytotoxicity towards the three cancer cells lines. The pH-response anti-proliferation effect of Ru@SiO_2_-PA was further confirmed in human normal cells, and the result indicated that Ru@SiO_2_-PA under pH 6.0 environment showed better anti-proliferation effect against HUVECs than that of pH 7.0 environment. Ru@SiO_2_-PA-induced anticancer activity was further confirmed by Giemsa staining. As shown in Fig. 3b, Ru@SiO_2_-PA treatment dose-dependently inhibited SGC-7901 cells growth, as convinced by the decreased cells number. Ru@SiO_2_ treatment only caused slight change of SGC-7901 cells number. Taken together, our findings validated the rational design that ruthenium-loaded PA-modified mesoporous silica (Ru@SiO_2_-PA) can act as novel nano-Fenton reactors to achieve tumor microenvironment-response anticancer activity.

### Ru@SiO_2_-PA induces enhanced cell apoptosis

**Fig. 4:**
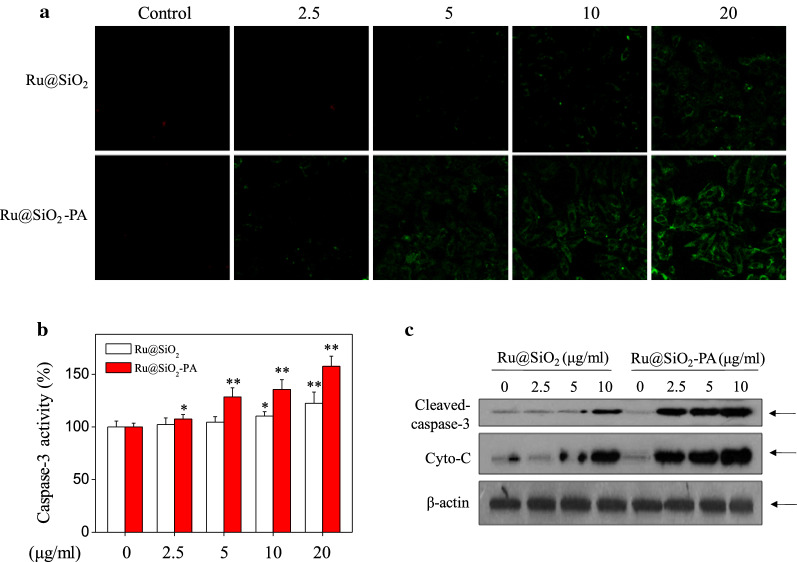
Ru@SiO_2_-PA induced enhanced cells apoptosis. SGC-7901 cells seeded in 6-well plate were treated with 2.5, 5, 10 and 20 μg/ml Ru@SiO_2_ or Ru@SiO_2_-PA for 6 h. Cells after treatment were loaded with AnnexinV probe and the early apoptosis was examined by fluorescent microscope (magnification, ×200). **b** Ru@SiO_2_-PA induced enhanced caspase-3 activation. SGC-7901 cells after treatment with Ru@SiO_2_ or Ru@SiO_2_-PA for 72 h were collected and total protein was extracted. Caspase-3 activity was detected with a specific Ac-DEVD-AMC substrate by fluorescent microreader. **c** Ru@SiO_2_-PA induced enhanced caspase-3 cleavage and cytochrome-c release. SGC-7901 cells after treatment with Ru@SiO_2_ or Ru@SiO_2_-PA for 72 h were collected and total protein was extracted. Protein expression was detected by western bolt analysis. Bars with “*” or “**” represents P < 0.05 or P < 0.01 level, respectively

MTT results showed that Ru@SiO_2_-PA caused more cytotoxicity towards SGC-7901 cells, which herein were selected for further mechanism study. The extracellular ectropion of phosphatidylserine protein is usually considered as an index for cells early apoptosis [30]. Therefore, Ru@SiO_2_-PA-induced early apoptosis was primary detected by annexin V probe. As shown in Fig. 4a, Ru@SiO_2_ treatment caused slight early apoptosis of SGC-7901 cells, as convinced by the green fluorescence. However, Ru@SiO_2_-PA treatment dose-dependently triggered enhanced early apoptosis of SGC-7901 cells, as convinced by the enhanced green fluorescence. Caspase-3 as the main apoptosis executor was also detected, and the result indicated that Ru@SiO_2_-PA treatment caused more activation of caspase-3 than that of Ru@SiO_2_ (Fig. 4b). Caspase-3 expression by western blot analysis further confirmed Ru@SiO_2_-PA-induced enhanced apoptosis of SGC-7901 cells (Fig. 4c). Moreover, Ru@SiO_2_-PA treatment also dose-dependently caused enhanced cytochrome-c release (Fig. 4c), which will irreversibly lead to apoptosis of SGC-7901 cells. Taken together, these results suggested that Ru@SiO_2_-PA after PA modification induced enhanced cancer cells apoptosis.

### Enhanced inhibitory effects on tumor spheroids

**Fig. 5:**
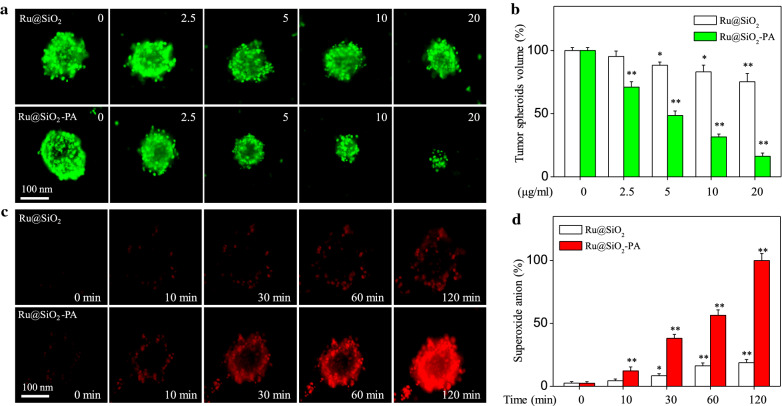
Enhanced inhibitory effect on tumor spheroids. **a** Dose-dependent inhibitory effect of Ru@SiO_2_ or Ru@SiO_2_-PA on tumor spheroids. SGC-7901 cells (6 × 10^5^ cells/well) were seeded in ultra-low attachment 6-well plate and cultured for 2 days. Then, tumor spheroids were treated with Ru@SiO_2_ or Ru@SiO_2_-PA for 72 h, and imaged under a fluorescent microscope (magnification, 100 ×). **b** Quantitative analysis of tumor spheroids volume. **c** Real-time images of superoxide anion. SGC-7901 tumor spheroids cultured for 5 days were pre-incubated with DHE probe and treated 20 μg/ml Ru@SiO_2_ or Ru@SiO_2_-PA for 10–120 min. The Real-time generation of superoxide anion was imaged under a fluorescent microscope (magnification, ×100). **d** Quantitative analysis of superoxide anion. Bars with “*” or “**” represents P < 0.05 or P < 0.01 level, respectively

To further confirm Ru@SiO_2_-PA-induced anticancer efficiency, 3D tumor spheroids of SGC-7901 cells were established in the present study, which was considered as a good tumor model in ex vivo. As shown in Fig. 5a, Ru@SiO_2_ treatment slightly inhibited the growth of SGC-7901 tumor spheroids. However, Ru@SiO_2_-PA treatment dose-dependently induced enhanced growth inhibition against SGC-7901 tumor spheroids, as convinced by the decreased volume of tumor spheroids. The quantitative analysis of tumor spheroids volume further confirmed this inhibitory effect (Fig. 5b). For instance, Ru@SiO_2_ (5, 10 and 20 μg/ml) only inhibited the volume of SGC-7901 tumor spheroids from 100% (control) to 80.1%, 75% and 68.2%, respectively. However, Ru@SiO_2_-PA (5, 10 and 20 μg/ml) effectively inhibited the volume of SGC-7901 tumor spheroids from 100% (control) to 51.2%, 37.2% and 17.5%, respectively. Taken together, these results demonstrated that Ru@SiO_2_-PA after PA modification induced enhanced growth inhibition against SGC-7901 tumor spheroids in ex vivo.

### Ru@SiO_2_-PA inhibits tumor xenografts growth in vivo

**Fig. 6:**
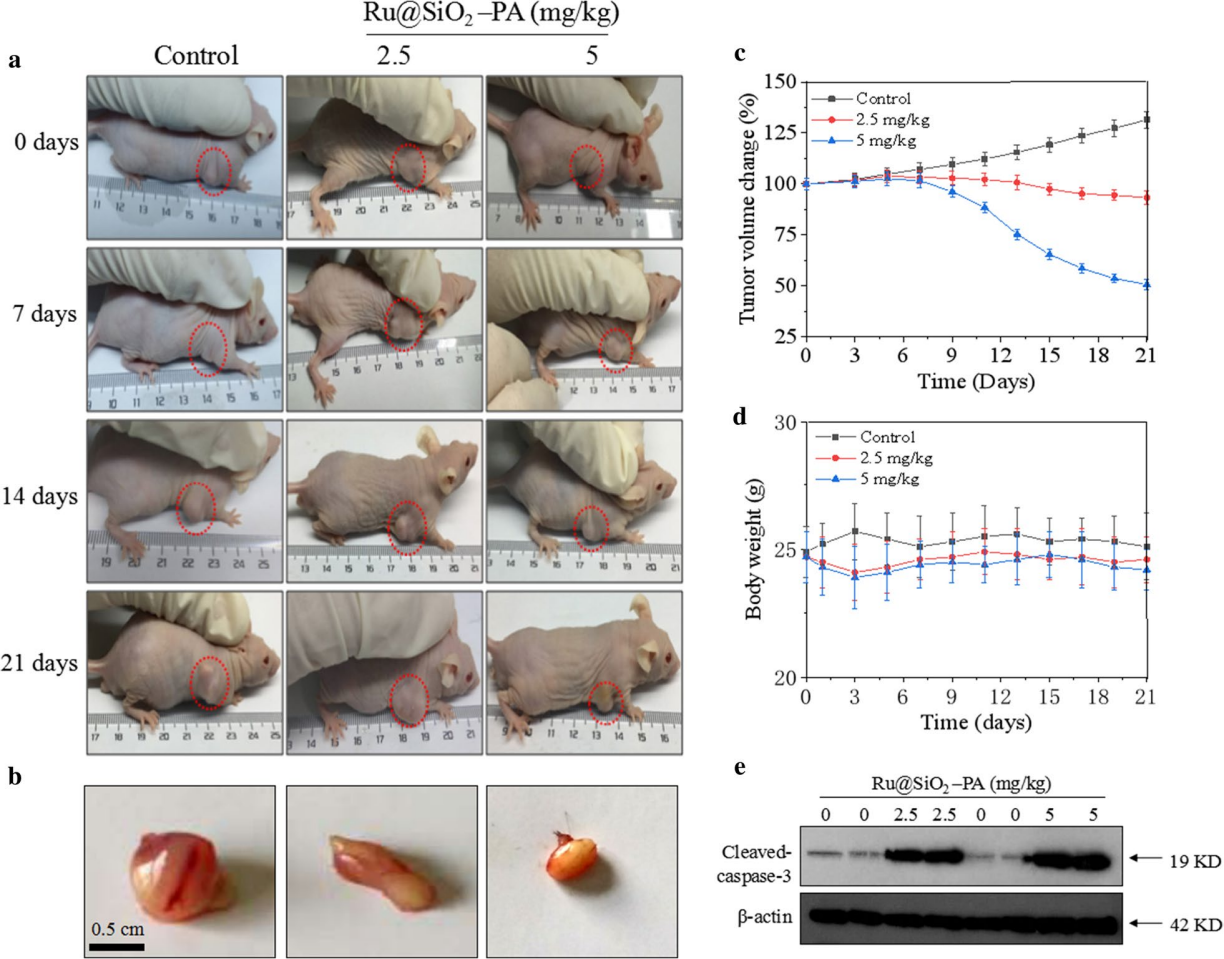
Ru@SiO_2_-PA inhibited SGC-7901 tumor growth in vivo. **a** Images of nude mice bearing SGC-7901 tumor xenografts. **b** Tumor images. **c** Real-time change curve of tumor volume. **d** Real-time change curve of body weight. SGC-7901 cells (10^7^ cells) were subcutaneously injected into the nude mice. After 7-days growth, mice were given 2.5 and 5 mg/kg Ru@SiO_2_-PA every other day for 21 days. The nude mice bearing SGC-7901 tumor xenografts were imaged. The real-time change curve of tumor volume and mice body weight were measured. **e** Ru@SiO_2_-PA induced caspase-3 activation in vivo. Caspase-3 expression was detected by western blot analysis. All experiments were repeated at least 3 times. Bars with “**” represent P < 0.01 level

To evaluate Ru@SiO_2_-PA-induced anticancer efficiency in vivo, nude mice bearing SGC-7901 tumor xenografts were employed in the present study. Nude mice were planted with SGC-7901 cells (10^7^ cells) by subcutaneous injection, and nude mice after 21-days growth showed notable tumors (Fig. 6a). Nude mice were administrated with 5 and 10 mg/kg Ru@SiO_2_-PA for 21 days by tail intravenous injection every other day, and the results showed that Ru@SiO_2_-PA administration effectively inhibited SGC-7901 tumor xenografts in vivo, as convinced by the decreased tumor volume (Fig. 6b). The real-time change curve of tumor volume (Fig. 6c) further confirmed Ru@SiO_2_-PA-induced anticancer efficiency in vivo. Ru@SiO_2_-PA administration in vivo caused no significant change of mice body weight (Fig. 6d). The anticancer mechanism of Ru@SiO_2_-PA in vivo was also explored. As shown in Fig. 6e, Ru@SiO_2_-PA administration significantly caused caspase-3 activation, indicating that Ru@SiO_2_-PA induced tumor cells apoptosis in vivo. Taken together, these results demonstrated that Ru@SiO_2_-PA had the potential to inhibit tumor xenografts growth in vivo by induction of apoptosis.

### Biodistribution of Ru@SiO_2_-PA in vitro and in vivo

**Fig. 7:**
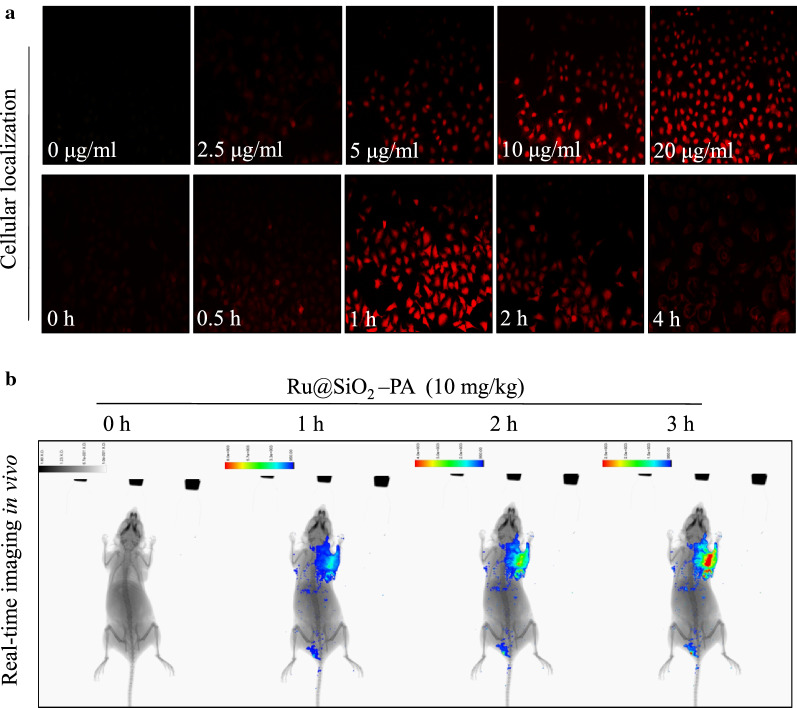
Biodistribution of Ru@SiO_2_-PA in vitro and in vivo. **a** Dose-dependent and time-dependent imaging in vitro. SGC-7901 cells were treated with 0–20 μg/ml Ru@SiO_2_-PA for 4 h, or cells were treated with 10 μg/ml Ru@SiO_2_-PA for 0–4 h. The biodistribution of Ru@SiO_2_-PA in vitro were imaged by measure the Ru fluorescence. **b** Real-time imaging in vivo. The in vivo biodistribution of Ru@SiO_2_-PA was monitored by real-time imaging in nude mice. Ru@SiO_2_-PA (10 mg/kg) was given by tail vein injection, and real-time imaging of Ru fluorescence at 1, 2 and 3 h was detected by in vivo fluorescence imaging system (Caliper Perkin Elmer)

Studies have verified that ruthenium can induce DNA-targeted incorporation and emit bright red fluorescence, which can be used to monitor the real-time biodistribution of Ru@SiO_2_-PA in vitro and in vivo. As shown in Fig. 7a, SGC-7901 cells exposed to 2.5 μg/ml Ru@SiO_2_-PA showed time-dependent red fluorescence. The real-time imaging of Ru@SiO_2_-PA in SGC-7901 cells vividly convinced that the biodistribution of Ru@SiO_2_-PA in vitro was mainly at nucleus. What’s more, the biodistribution of Ru@SiO_2_-PA in vivo was also monitored in nude mice by fluorescence imaging system. As shown in Fig. 7b, nude mice after tail intravenous injection of 10 mg/kg Ru@SiO_2_-PA showed obvious fluorescence with a time-dependent manner. The real-time imaging of Ru@SiO_2_-PA in nude mice vividly convinced that the biodistribution of Ru@SiO_2_-PA in vivo was mainly at SGC-7901 tumor xenografts. Taken together, these results validated that ruthenium loading endowed Ru@SiO_2_-PA with real-time monitoring property.

### Ru@SiO_2_-PA causes enhanced dysfunction of MAPKs and PI3K/AKT pathways

**Fig. 8:**
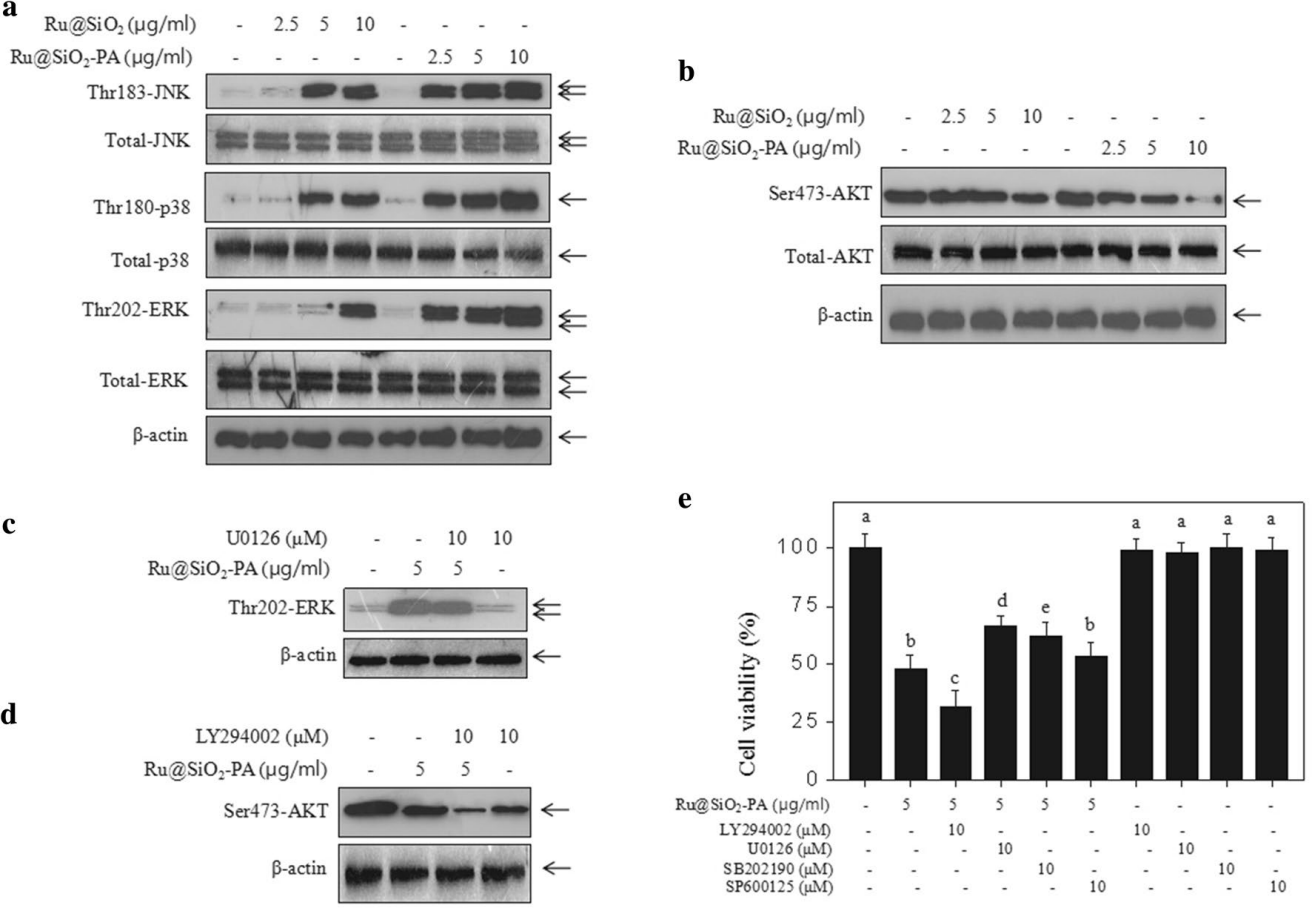
Enhanced dysfunction of MAPKs and PI3K/AKT pathways. **a** Dose-dependent dysfunction of MAPKs pathway. **b** Dose-dependent dysfunction of PI3K/AKT pathway. **c** Effect of U0126 (ERK inhibitor) on p-ERK expression in Ru@SiO_2_-PA-treated cells. **d** Effect of LY294002 (AKT inhibitor) on p-AKT expression in Ru@SiO_2_-PA-treated cells. **e** Effects of four inhibitors on Ru@SiO_2_-PA-treated cells viability. SGC-7901 cells were pre-treated with inhibitors for 2 h or/and co-treated with 2.5–10 μg/ml Ru@SiO_2_ or Ru@SiO_2_-PA for 72 h. Protein expression was examined by western blot analysis. Cells viability was detected by MTT assay. Bars with different letters indicate the P < 0.05 level

MAPKs (including p38, JNK and ERK) and PI3K/AKT pathways are the two most important pathways in regulating cells proliferation, cell growth and cell apoptosis [31–33]. ROS-mediated oxidative stress as an early apoptotic event was located at the upstream of MAPKs and PI3K/AKT pathways. Studies have verified that dysfunction of MAPKs and PI3K/AKT pathways both contributed to nano-drugs-induced anticancer mechanisms [34–36]. Hence, the status of MAPKs and PI3K/AKT pathways in Ru@SiO_2_-PA-treated SGC-7901 cells was evaluated by western blot analysis. As shown in Fig. 8a, b, Ru@SiO_2_ treatment slightly activated MAPKs and inactivated PI3K/AKT pathway in SGC-7901 cells. However, Ru@SiO_2_-PA treatment dose-dependently induced enhanced activation of MAPKs and inactivation of PI3K/AKT pathway, as convinced by the enhanced phosphorylation level of JNK, p38 and ERK, and decreased phosphorylation level of AKT, respectively (Fig. 8a, b). To further confirm the significance of MAPKs and PI3K/AKT pathways, four inhibitors of the two pathways were employed. The results indicated that ERK inhibitor by U0126 significantly attenuated Ru@SiO_2_-PA-induced ERK activation (Fig. 8c). Meanwhile, AKT inhibitor by LY294002 significantly enhanced Ru@SiO_2_-PA-induced AKT inactivation (Fig. 8d). Effects of four inhibitors on cells viability further confirmed this conclusion (Fig. 8e). As shown in Fig. 8e, pre-treatment of SGC-7901 cells with LY294002 (AKT inhibitor) significantly enhanced Ru@SiO_2_-PA-induced growth inhibition against SGC-7901 cells. Pre-treatment of SGC-7901 cells with U0126 (ERK inhibitor) or SB202190 (p38 inhibitor) both significantly attenuated Ru@SiO_2_-PA-induced growth inhibition against SGC-7901 cells (Fig. 8e). Pre-treatment of SGC-7901 cells with SP600125 (JNK inhibitor) caused no significant change of Ru@SiO_2_-PA-induced cell growth. Taken together, these results suggested that Ru@SiO_2_-PA inhibited anticancer cells growth with MAPKs- and PI3K/AKT-dependent manner, which validated that Ru@SiO_2_-PA after PA modification caused enhanced dysfunction of MAPKs and PI3K/AKT pathways.

### Ru@SiO_2_-PA triggers enhanced oxidative damage in vitro and ex vivo

**Fig. 9:**
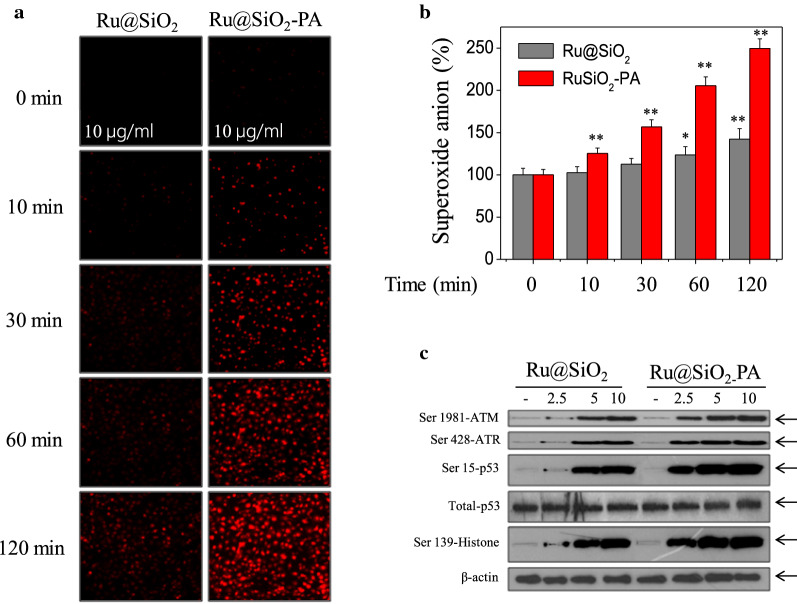
Ru@SiO_2_-PA triggers enhanced oxidative damage. **a** Real-time imaging of superoxide anion. SGC-7901 cells were pre-loaded with 10 μM DHE probe for 15 min in darkness, and treated with 10 μg/ml Ru@SiO_2_ or Ru@SiO_2_-PA for 10–120 min. The real-time generation of superoxide anion was imaged under a fluorescent microscope (magnification, ×100). **b** Quantitative analysis of superoxide anion. Generation of superoxide anion was quantified as % of the control. **c** Ru@SiO_2_-PA triggered enhanced DNA damage. Several DNA damaging markers were examined by western blot analysis. Bars with “*” or “**” represents P < 0.05 or P < 0.01 level, respectively

**Fig. 10:**
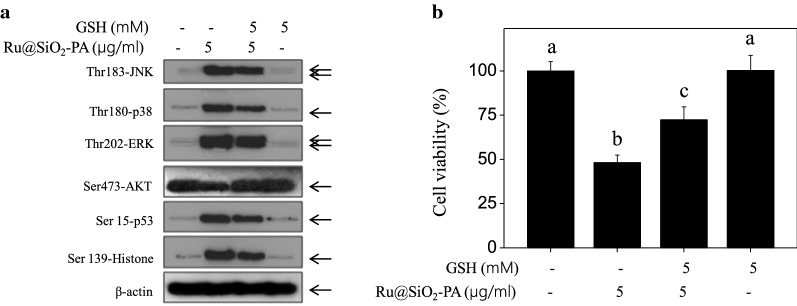
Inhibition of ROS decreased Ru@SiO_2_-PA-induced anticancer activity. **a** Inhibition of ROS by glutathione (GSH) inhibited Ru@SiO_2_-PA-induced dysfunction of MAPKs and PI3K/AKT pathways and DNA damage. **b** Inhibition of ROS inhibited Ru@SiO_2_-PA-induced anticancer activity. SGC-7901 cells were pre-treated with 5 mM GSH for 2 h and co-treated with 5 μg/ml Ru@SiO_2_-PA for 72 h. Protein expression was examined by western blot analysis. Cells viability was assayed by MTT assay. Bars with different letters indicate the P < 0.05 level

H_2_O_2_ in acid tumor microenvironment was generated by Fenton reaction in the presence of PA, and H_2_O_2_ can be reduced by Ru^2+^ into ·OH, which will cause oxidative damage [10, 26, 27]. Herein, Ru@SiO_2_-PA-induced oxidative damage was examined in vitro and *ex *in vivo. As shown in Fig. 9a, Ru@SiO_2_ treatment slightly induced the generation of superoxide anion. However, Ru@SiO_2_-PA treatment time-dependently induced enhanced generation of superoxide anion in vitro, as convinced by the enhanced red fluorescence. The quantitative analysis of superoxide anion accumulation further confirmed this effect (Fig. 9b). The enhanced generation of superoxide anion was also detected in tumor spheroids ex vivo (Fig. 5c, d). Overproduction of superoxide anion will trigger DNA damage. As shown in Fig. 9c, Ru@SiO_2_ treatment slightly triggered DNA damage. However, Ru@SiO_2_-PA treatment dose-dependently triggered enhanced DNA damage, as convinced by the enhanced phosphorylation level of ATM, ATR, p53 and histone. Taken together, these results validated the rational design that ruthenium-loaded PA-modified mesoporous silica (Ru@SiO_2_-PA) can act as novel nano-Fenton reactors to trigger tumor microenvironment-response oxidative damage in cancer cells (Fig. 10).

### Safety evaluation

**Fig. 11:**
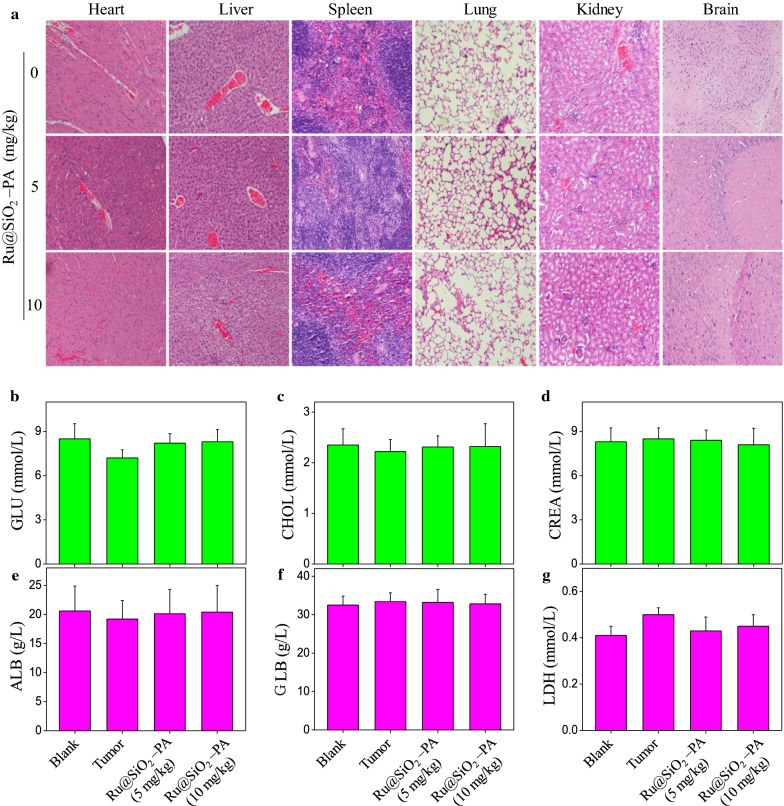
Analysis of pathology and hematology. **a** Histopathological examination. Nude mice after treatment were all given euthanasia, and main organs (heart, liver, spleen, lung, kidney and brain) were collected, cut into 4 μM slices and stained by H&E for histopathological examination under a microscope (magnification, ×100). **b** Hematological analysis. Blood from nude mice or nude mice bearing SGC-7901 tumor xenografts was collected, and blood glucose (GLU), cholesterol (CHOL), serum creatinine (CREA), albumen (ALB), globulin (GLB) and lactate dehydrogenase (LDH) were all detected by ELISA methods

Drugs safety was an important index for anticancer drugs design. Herein, side effects of Ru@SiO_2_-PA were examined in vitro and in vivo. As shown in Fig. 3a, Ru@SiO_2_-PA under pH 6.0 and pH 7.4 showed distinct cells killing. That is, Ru@SiO_2_-PA showed less toxicity towards human normal cells in vitro, which validated that Ru@SiO_2_-PA as novel nano-Fenton reactors displayed super selectivity between cancer cells and normal cells. The in vivo side effects of Ru@SiO_2_-PA in mice were also examined. Nude mice after administration with 5 and 10 mg/kg Ru@SiO_2_-PA for 21 days was given euthanasia, and main organs were collected, cut into 4 μM slices and stained by H&E for histopathological examination. As shown in Fig. 11a, Ru@SiO_2_-PA administration in vivo caused no obvious impairment and inflammation of heart, liver, spleen, lung, kidney and brain compared to that of the control groups. Moreover, blood biochemical indexes were also assayed by ELISA methods. As shown in Fig. 11b–g, nude mice after Ru@SiO_2_-PA administration showed no significant changes of blood glucose (GLU), cholesterol (CHOL), serum creatinine (CREA), albumen (ALB), globulin (GLB) and lactate dehydrogenase (LDH), indicating the health of kidney, liver and heart. Taken together, these results indicated the safety of Ru@SiO_2_-PA in vitro and in vivo with potential application in clinic.

## Conclusion

In the present study, ruthenium-loaded palmitoyl ascorbate (PA)-modified mesoporous silica (Ru@SiO_2_-PA) was successfully fabricated and characterized, and the results indicated that Ru@SiO_2_-PA under pH6.0 environment displayed enhanced growth inhibition and apoptosis against human cancer cells in vitro and in vivo. Mechanism investigation revealed that Ru@SiO_2_-PA triggered ROS-mediated DNA damage and dysfunction of MAPKs and PI3K/AKT pathways. Importantly, Ru@SiO_2_-PA showed no side effects in vivo, which validated the rational design that ruthenium-loaded PA-modified mesoporous silica (Ru@SiO_2_-PA) can act as novel tumor microenvironment-response nano-Fenton reactors for cancer precise therapy.

## Data Availability

The datasets used and/or analyzed during the current study are available within the manuscript.
